# Solitomab, an EpCAM/CD3 bispecific antibody construct (BiTE®), is highly active against primary uterine and ovarian carcinosarcoma cell lines *in vitro*

**DOI:** 10.1186/s13046-015-0241-7

**Published:** 2015-10-17

**Authors:** Francesca Ferrari, Stefania Bellone, Jonathan Black, Carlton L. Schwab, Salvatore Lopez, Emiliano Cocco, Elena Bonazzoli, Federica Predolini, Gulden Menderes, Babak Litkouhi, Elena Ratner, Dan-Arin Silasi, Masoud Azodi, Peter E. Schwartz, Alessandro D. Santin

**Affiliations:** Department of Obstetrics, Gynecology & Reproductive Sciences, Yale University School of Medicine, New Haven, CT USA; 333 Cedar Street, LSOG 305, PO Box 208063, New Haven, CT 06520-8063 USA

**Keywords:** EpCAM, CD3, T-lymphocyte, Bispecific antibody, Uterine carcinosarcoma, Ovarian carcinosarcoma

## Abstract

**Background:**

Uterine and ovarian carcinosarcomas (CS) are rare but highly aggressive gynecologic tumors which carry an extremely poor prognosis. We evaluated the expression levels of EpCAM and the *in vitro* activity of solitomab, a bispecific single-chain antibody construct which targets epithelial-cell-adhesion-molecule (EpCAM) on tumor cells and also contains a CD3 binding region, against primary uterine and ovarian CS cell lines.

**Methods:**

EpCAM expression was evaluated by flow cytometry in a total of 5 primary CS cell lines. Sensitivity to solitomab-dependent-cellular-cytotoxicity (ADCC) was tested against the panel of primary CS cell lines expressing different levels of EpCAM in standard 4 h ^51^Cr release-assays. The proliferative activity, activation, cytokine secretion (i.e., Type I vs Type II) and cytotoxicity of solitomab in autologous tumor-associated-T cells (TAL) in the pleural fluid of a CS patient were also evaluated by CFSE and flow-cytometry assays.

**Results:**

Surface expression of EpCAM was found in 80.0 % (4 out of 5) of the CS cell lines tested by flow cytometry. EpCAM positive cell lines were found resistant to NK or T-cell-mediated killing after exposure to peripheral blood lymphocytes (PBL) in 4-h chromium-release assays (mean killing ± SEM = 1.1 ± 1.6 %, range 0–5.3 % after incubation of EpCAM positive cell lines with control BiTE®). In contrast, after incubation with solitomab, EpCAM positive CS cells became highly sensitive to T-cell-cytotoxicity (mean killing ± SEM of 19.7 ± 6.3 %; range 10.0-32.0 %; *P* < 0.0001). Ex vivo incubation of autologous TAL with EpCAM expressing malignant cells in pleural effusion with solitomab, resulted in a significant increase in T-cell proliferation in both CD4+ and CD8+ T cells, increase in T-cell activation markers (i.e., CD25 and HLA-DR), and a reduction in number of viable CS cells in the exudate (*P* < 0.001).

**Conclusions:**

Solitomab may represent an effective treatment for patients with recurrent/metastatic and/or chemo-resistant CS overexpressing EpCAM.

## Introduction

Gynecological carcinosarcomas (CS), also known as Malignant Mixed Mullerian Tumors (MMMT), are rare biphasic tumors that most commonly arise in the uterus and ovaries [1]. Histologically, the carcinomatous component may include endometrioid, serous, clear cell, squamous or mucinous differentiation or may be undifferentiated. CS are classified on the basis of the nature of their mesenchymal elements as "homologous" when mesenchymal components differentiate towards tissues physiologically native to the primary site (e.g. leiomyosarcoma, fibrosarcoma and endometrial stromal sarcoma), or as “heterologous” when they contain mesenchymal components that are physiologically foreign to the primary site (e.g. chondrosarcoma, osteosarcoma, rhabdomyosarcoma and liposarcoma). While the pathogenesis of CS remains under debate, an increasing body of evidence supports the origin of both elements from a common epithelial cell that undergoes sarcomatous dedifferentiation, rather than two independent progenitors [2–5]. While CS comprise only a small percentage (i.e., 1 to 5 %) of ovarian and uterine cancers, the overall 5 year survival for ovarian and uterine CS patient remains a dismal 24 % and 36 %, respectively [1]. The discovery of novel diagnostic and therapeutic markers against this aggressive subset of gynecological tumors remains a high priority.

Overexpression of epithelial cell adhesion molecule-1 (EpCAM), also known as TROP-1 or TACSTD1, is a known poor prognostic biomarker across a large number of carcinomas and carcinosarcomas [4, 5]. EpCAM, a 39 to 42 kd protein, consists of three domains including; an extracellular domain with 2 epidermal growth factor-like repeats, a transmembrane domain, and a short 26 amino acid cytoplasmic domain. EpCAM promotes cell adhesion and is expressed at low levels on different various epithelia. It is predominantly expressed in the basolateral and intercellular surface of simple, pseudo-stratified, and transitional epithelia as well as most epithelial tissues in the female genital tract [6]. EpCAM is known to play an important role in multiple cell functions including cell migration, cell signaling, differentiation, and proliferation. High levels of expression on the cell surface of multiple human carcinomas makes EpCAM an attractive target for immunotherapy [7]. The discovery of EpCAM (CD326) expression in a number of solid tumors led to the development of Solitomab (MT110, AMG 110) which is an EpCAM/CD3-bispecific single-chain antibody construct [8]. Solitomab acts by engaging resting polyclonal CD8+ and CD4+ T cells for highly potent redirected lysis of target tumor cells that express EpCAM. Solitomab’s antitumor activity in preclinical EpCAM positive ovarian tumor xenograft models has shown promise. In the current study, we have used flow cytometry and q-Real-time-PCR to evaluate EpCAM expression in primary carcinosarcomas cell lines and explored for the first time the potential of solitomab, an EpCAM/CD3 BiTE®, as a novel therapeutic strategy against carcinosarcoma cell lines and un-manipulated malignant tumor cells isolated from malignant pleural fluid of a patient. Our results demonstrate impressive solitomab antitumor activity against carcinosarcoma cell lines and tumor cells in pleural fluid from patients with gynecologic carcinosarcomas.

## Methods

### Patients and sample processing

All patients signed an informed consent form according to institutional guidelines and approval for this in vitro study was obtained from the institutional review board. A total of five primary carcinoma cell lines [i.e., two primary uterine carcinosarcoma cell lines (SARARK-1 and SARARK-9) and three primary ovarian carcinosarcoma cell lines (SARARK-3, SARARK-6 and SARARK-7)] were established after sterile processing of surgical biopsy specimens, as described previously [9]. Briefly, tumor tissue was mechanically minced to portions no larger than 1 to 3 mm3 in an enzyme solution made of 0.14 % collagenase type I (Sigma) and 0.01 % DNase (Sigma, 2000 KU/mg) in RPMI 1640, and incubated in the same solution in a magnetic stirring apparatus for an hour at room temperature. Enzymatically dissociated cells were then washed twice in RPMI 1640 with 10 % fetal bovine serum and maintained in RPMI supplemented with 10 % fetal bovine serum, 200 μg/ml of penicillin and 200 μg/ml of streptomycin at 37 °C, 5 % CO2 in 75 cm2 tissue culture flasks or Petri dishes (Corning). After seeding on plasticware for 48–72 h, nonadherent cells and contaminant inflammatory cells were gently removed from the culture by multiple washings with PBS. Both primary uterine carcinosarcoma cell lines were established from biopsies of the uterus of chemotherapy naïve patients at the time of the primary staging surgery, while all primary ovarian carcinosarcoma cell lines were obtained from the biopsy of metastatic sites of disease in patients harboring recurrent, chemotherapy-resistant disease. In all primary ovarian carcinosarcoma cell lines cases, the high *in vivo* resistance to multiple chemotherapy agents was confirmed *in vitro* by MTT chemotherapy resistance assays against multiple cytotoxic agents (data not shown). Primary carcinosarcoma cell lines were tested for presence of EpCAM by Quantitative Real-time PCR and by flow cytometry as described below. An additional tumor sample was collected from a CS patient with recurrent disease and a large pleural effusion. The fluid sample was cytologically confirmed to contain a large number of EpCAM + carcinosarcoma cells at the time of a therapeutic thoracentesis. The fresh sample of pleural fluid was plated into 6-well microtiter plate for treatment using solitomab and a nonspecific BiTE® control antibody construct without prior processing. Cell numbers and viability were determined by flow cytometry as described below. Patient characteristics of all carcinosarcoma cell lines and the pleural fluid exudate are described in Table 1.

**Table 1:** Patient characteristics and EpCAM Protein Expression by Flow Cytometry and by qReal-Time PCR in carcinosarcoma cell lines

Cell Line	Histology		Age	Race	FIGO Stage	Primary site	Percentage EpCAM-Positive Cells	MFI	Avg dCt qReal-Time PCR
	ES	SC							
SARARK-1	Homologous	ESS	70	AA*	IC	Uterus	10.6	13.2	17.87
	END + CC								
SARARK-3	Heterologous	CDRS	75	C	IIIC	Ovary	100	726	3.84
	SER								
SARARK-6	Homologous	CDR	78	C	IV/IIB	Ovary	100	301.2	3.57
	SER								
SARARK-7	Heterologous	CDRS	55	AA	IV	Ovary	100	939.9	7.16
	CC + SER								
SARARK-9	Homologous	ESS	66	C	IIIC2	Uterus	100	862.6	6.05
	SER								

*AA African-American, *C* Caucasian, *FIGO* International Federation of Gynecology and Obstetrics, *EC* epithelial component, *SC* stromal component, *END* endometrioid, *ESS* endometrial stromal sarcoma, *CC* clear cell, *CDR* chondroid, *CDRS* chondrosarcoma, *SER* serous

### Ex vivo therapy of malignant pleural fluid sample

Malignant fluid sample was analyzed after *ex vivo* treatment with solitomab or a control bispecific antibody construct. Briefly, the malignant fluid sample was plated in duplicate in 6-well flat microtiter plate. The pleural fluid was treated with the bispecific antibody construct, solitomab (Amgen Research Munich GmbH, Munich, Germany) at a concentration of 1 μg/ml for 7 days. In control wells, pleural fluid was treated with control BiTE® huMEC14 also at a concentration of 1 μg/ml. The effect of solitomab on the malignant tumor cells was assessed by observation of induction of morphologic changes and extent of cytotoxicity, as well as, for evidence of T cell activation and induction of cytokine release as described below.

### Quantitative real-time polymerase chain reaction

RNA isolation from all five primary carcinosarcoma cell lines were performed using TRIzol Reagent (Invitrogen) according to the manufacturer’s instructions as previously described. The endogenous control, glyceraldehyde-3-phosfate dehydrogenase (GAPDH) Assay Hs99999905_ml (Applied Biosystems, Foster City, CA) was used to normalize variations in cDNA quantities from different samples. The comparative threshold cycle (C_T_) method was used for the calculation of amplification fold as specified by the manufacturer. Quantitative real-time PCR (qRT-PCR) was done with a 7500 Real-time PCR System using the protocols recommended by the manufacturer (Applied Biosystems) to evaluate expression of EpCAM in all samples. Briefly, 5 μg of total RNA from each sample was reverse transcribed using SuperScript III first-strand cDNA synthesis (Invitrogen). Five μl of reverse transcribed RNA samples (from 500 μl of total volume) were amplified by using the TaqMan Universal PCR Master Mix (Applied Biosystems) to produce PCR products specific for EpCAM. The C_T_ method (Applied Biosystems) was used to determine gene expression in each sample relative to the value observed in a control cell line known to express EpCAM, using GAPDH (Assay ID Hs99999905_ml) RNA as internal controls.

### Flow cytometry

Characterization of EpCAM expression in primary uterine and ovarian carcinosarcoma cell lines was performed by FACS analysis. The anti-human EpCAM-PE antibody clone 1B7 (eBioscience) was used for flow cytometry studies. The IgG1-PE antibody (BD Biosciences) was used as antibody isotype control for the anti-EpCAM antibody. Moreover a Human recombinant IgG1 anti-EpCAM monoclonal antibody (mAb) MT201 (Micromet AG) was used for flow cytometry studies. Briefly, cell lines were stained with MT201 (Micromet AG). The chimeric anti-CD20 mAb rituximab (Rituxan, Genentech, San Francisco, CA) was used as a control. A goat antihuman F(ab′)_2_ immunoglobulin (BioSource International, Camarillo, CA) was used as a secondary reagent. Analysis was conducted with FACScalibur flow cytometer with Cell Quest software (Becton Dickinson, Franklin lakes, NJ).

### T cell stimulation assay

Solitomab induced T cell activation was measured by detecting CD25 protein surface expression and HLA-DR expression on CD8^+^ and CD4^+^ T cells by FACS. Solitomab mediated stimulation of T cells was calculated according to the following formula: Percentage of CD8^+^/CD25^+^ expression = [number of CD8^+^/CD25^+^ cells/ total number of CD8^+^ cells] x 100. Similarly, using the same equation the number of CD8^+^/HLA-DR^+^, CD4^+^/CD25^+^ and CD4^+^/HLA-DR^+^ expression was calculated.

### Cytokine analysis

The level of solitomab dependent cytokine induction was compared to the corresponding value of percentage of cytokine released in the non-specific antibody control wells. This was performed by treating the solitomab and control non-specific antibody construct wells with phorbol myristate acetate and ionomycin followed by a 3 h incubation period to allow for lymphocyte stimulation. Brefeldin A was added and a further incubation for 3 h occurred in order to enhance intracellular cytokine staining signals. Cytokine analysis of the supernatants was performed by FACS analysis after adding anti-CD8-FITC antibody for surface staining followed by fixation, permeabilization and intracellular staining with anti-IL-4-PE antibody and anti-IFN gamma-PE antibody. Solitomab mediated release of each of these cytokines was calculated according to the following exemplary formula: Percentage of CD8^+^/IFN gamma containing cells = [number of CD8^+^/IFN gamma cells/total number of CD8^+^ cells] × 100. Similar calculations were performed for CD4^+^ T cells (i.e., gated CD3+/CD8- T cells).

### Tests for T cell mediated cytotoxicity

The standard 4-h chromium (^51^Cr) release assay was used to measure the cytotoxic reactivity of Ficoll-Hypaque-separated peripheral blood lymphocytes (PBLs) from several healthy donors against five carcinosarcoma cell lines at effector to target ratios (E:T) of 10:1 and 20:1. The release of ^51^Cr from target cells was measured as evidence of tumor cell lysis after exposure of the tumor cells to a concentration of 1 μg/ml of solitomab. The negative control conditions were the incubation of target cells alone or with PBL without BiTE® antibody construct. As a positive control condition, 1 % sodium dodecyl sulfate (SDS) was used to achieve complete lysis of target cells. The control BiTE® huMEC14 at 1 μg/ml was used as the negative control for solitomab in this bioassay. The control BiTE® antibody construct shared the CD3 binding arm with solitomab, but otherwise recognizes an herbicide as an irrelevant antigen instead of recognizing EpCAM. The percentage cytotoxicity of solitomab was calculated by the following formula: *% cytotoxicity = 100 X (E-S)/T-S)*, where *E* is the experimental release, *S* is the spontaneous release by effector cells, *T* is the maximum release by target cells lysed with 1 % SDS.

### Statistics

The paired *t* test was used to evaluate the differences in cellular cytotoxicity levels in primary tumor cell lines treated with solitomab versus control BiTE® huMEC14. An increase in T-cell activation and the release of cytokines were analyzed using the *t test* for paired data. Statistical analysis was performed using SPSS version 18. A *P*-value of < 0.05 was considered as the level of statistical significance.

## Results

### Epithelial cell adhesion molecule levels in primary carcinosarcoma cell lines

We used flow cytometry to obtain highly sensitive measurements of EpCAM surface expression in two primary uterine carcinosarcoma cell lines (SARARK-1 and SARARK-9) and three primary ovarian carcinosarcoma cell lines (SARARK-3, SARARK-6 and SARARK-7). Table 1 depicts the characteristics of the patients from which the samples were collected and shows the percentage of EpCAM positive cells and corresponding mean fluorescence intensity (MFI) in primary carcinosarcoma cell lines by flow cytometry. Among the primary cell lines, 4 out of 5 (80 %) were found to express EpCAM in 100 % of the tumor cells. The percentage of EpCAM-positive cells and EpCAM expression levels were high in SARARK-3, SARARK-6, SARARK-7 and SARARK-9. In contrast, low to negligible EpCAM expression was observed in SARARK-1 cells. EpCAM surface expression was also confirmed by qReal-Time PCR. As shown in Table 1, good correlation was found between q-Real-Time PCR and flow cytometry data results.

#### Carcinosarcoma primary cell lines are resistant to natural killer (NK) cell activity but sensitive to solitomab-mediated T cell cytotoxicity

**Fig. 1:**
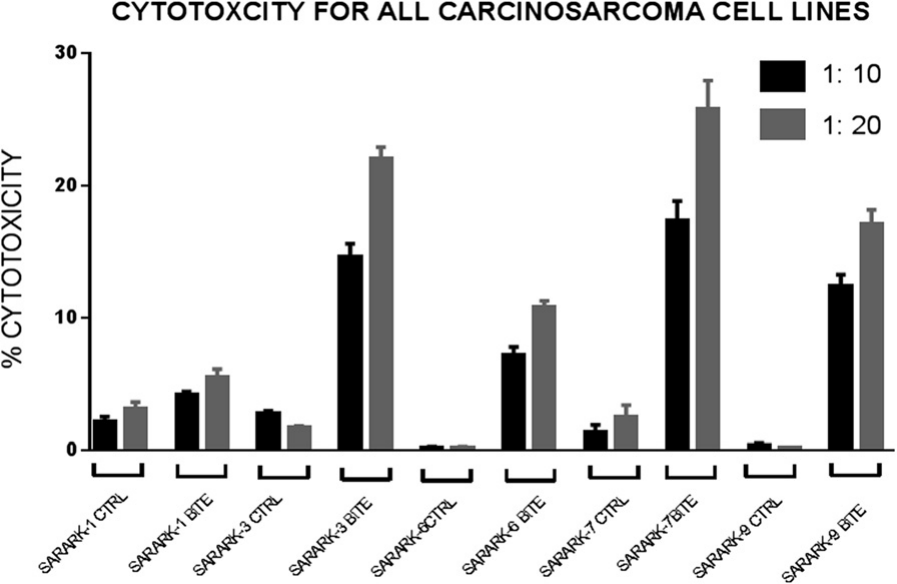
Graph showing antibody-dependent cell-mediated cytotoxicity for all five CS cell lines in control conditions and with solitomab at two effector: target ratios. The higher effector: target ratio of 1:20 exhibited more pronounced cytotoxicity results (EpCAM+ cell lines, ratio 1:10, *p* = 6.40731E-10, EpCAM+ cell lines, 1:20, *p* = 2.10467E-10). In EpCAM- cell line the difference in cytotoxicity between control Bite and solitumab was not significant)

We evaluated all five primary carcinosarcoma cancer cell lines for their sensitivity to NK and T-cells with a standard 4-h chromium (^51^Cr) release assay cytotoxicity using solitomab (EpCAM BiTE®) at a concentration of 1 μg/ml, as described previously [10]. The cell lines were exposed to peripheral blood lymphocytes collected from multiple healthy donors in several cytotoxicity assays. In this regard, peripheral blood lymphocytes typically contain between 20–25 % CD8^+^ T cells and 10–20 % NK cells (CD56^+^/CD16^+^). In previous titration experiments we found ADCC against tumor cells to plateau at 1 μg/ml concentration (10). This concentration was therefore used as optimal concentration in the cytotoxicity experiments described below. We found CS cell lines to be highly resistant to NK and T-cell-mediated killing when challenged with PBL at target: effector ratios of 1:10 and 1:20 (ratio 1:20, mean killing ± STDEV, 1.1 ± 1.6 % with range of killing 0–5.3 % after incubation of EpCAM-positive cell lines with control BiTE®) (Fig. 1). In contrast, significant killing was detected against all EpCAM-positive cell lines when challenged with PBL in the presence of solitomab at a ratio of killing of 20:1 (i.e., 10.0–32.0 %; mean killing ± STDEV, 19.7 ± 6.3 %; *P* < 0.0001). As expected, the low EpCAM expressing cell line (SARARK-1) demonstrated low to negligible levels of cytotoxicity when challenged with PBL in the presence of solitomab (Fig. 1).

#### Cytotoxicity evaluation in co-cultures of solitomab (EpCAM BiTE®) and CS tumor-associated lymphocytes

**Fig. 2:**
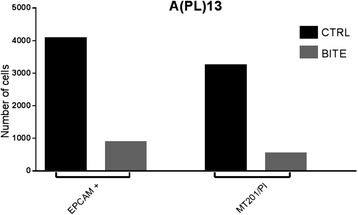
Graph showing the percentage of EpCAM positive viable cell in patients’ pleura exudate after 7 days incubation with control BiTE® vs solitomab. EpCAM (*p* = 2.09378E-06); MT201/P1 (*p* = 0.001724101) significant difference

Cytotoxicity was evaluated after co-culturing for 7 days EpCAM-positive CS tumor cells with solitomab and autologous TALs in a freshly collected pleural exudate fluid sample (APL13). Anti-EpCAM antibodies were used to label tumor cells to be counted by flow cytometry and human recombinant IgG1 anti-EpCAM monoclonal antibody (mAb) MT201 combined with propidium iodide (PI) for exclusion of dead cells. As shown in Fig. 2, incubation for 7 days with solitomab significantly decreased the number of EpCAM-positive tumor cells in the freshly collected pleural effusion sample.

#### Activation of T-lymphocytes by solitomab (EpCAM BiTE®) in co-cultures with carcinosarcoma cells

**Fig. 3:**
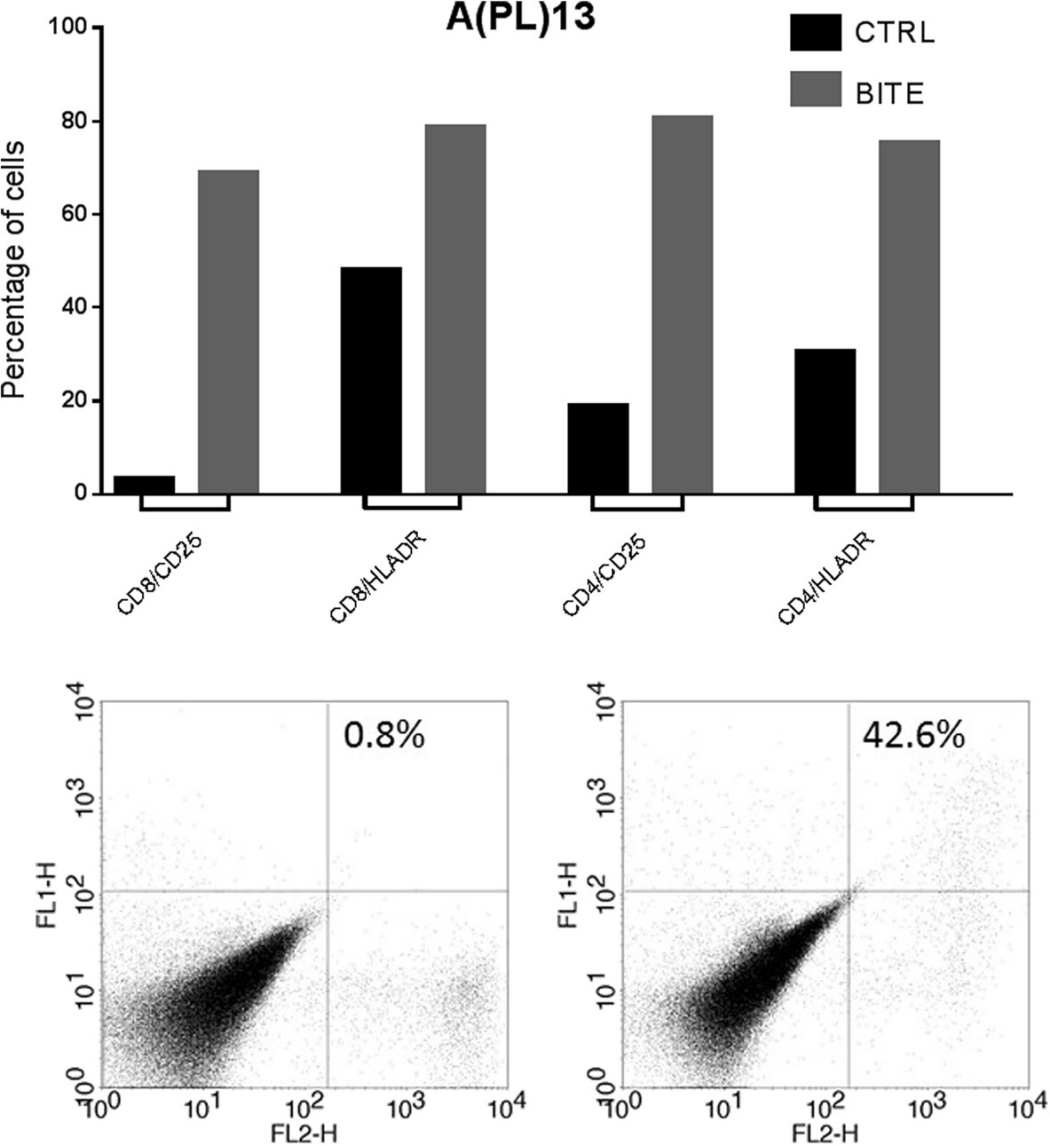
Upper panel: CD25 and HLA-DR activation marker expression in CD8^+^ and CD4^+^ T cells after stimulation of T-lymphocytes exposed to solitomab vs control BiTE®. A significant increase in CD25 and HLA-DR activation marker expression was consistently detected in both CD8^+^ or CD4^+^ T cells (CD8/CD25, CTRL VS BITE, *p* = 0.00000110594; CD8/HLADR, CTRL VS BITE *p* = 0.00017812290; CD4/CD25, CTRL VS BITE *p* = 0.00001948657; CD4/HLADR, CTRL VS BITE *p* = 0.00000732741). Lower panel: representative flow cytometry graphic evidencing the increase in the activation marker CD25 in CD8+ T lymphocytes after stimulation with Solitumab vs control BITE

Next, we explored whether solitomab is capable of inducing T cell activation in the presence of carcinosarcoma cells in minimally manipulated pleural fluid samples (i.e., APL13). CD8^+^ T and CD4^+^ T cells at baseline and after 7-days co-cultures with solitomab were analyzed by flow cytometry for the expression of the T-cell activation markers CD25 and HLA-DR. As demonstrated in the Fig. 3, we found a significant increase in both CD25 and HLA-DR protein surface expression on CD8^+^ and CD4^+^ T cells in the solitomab treated wells when compared to the wells treated with the control BiTE®. These data are consistent with our previous results with solitomab in the ascitic fluid of ovarian cancer patients [10].

#### Cytokine release accompanying T lymphocyte activation

**Fig. 4:**
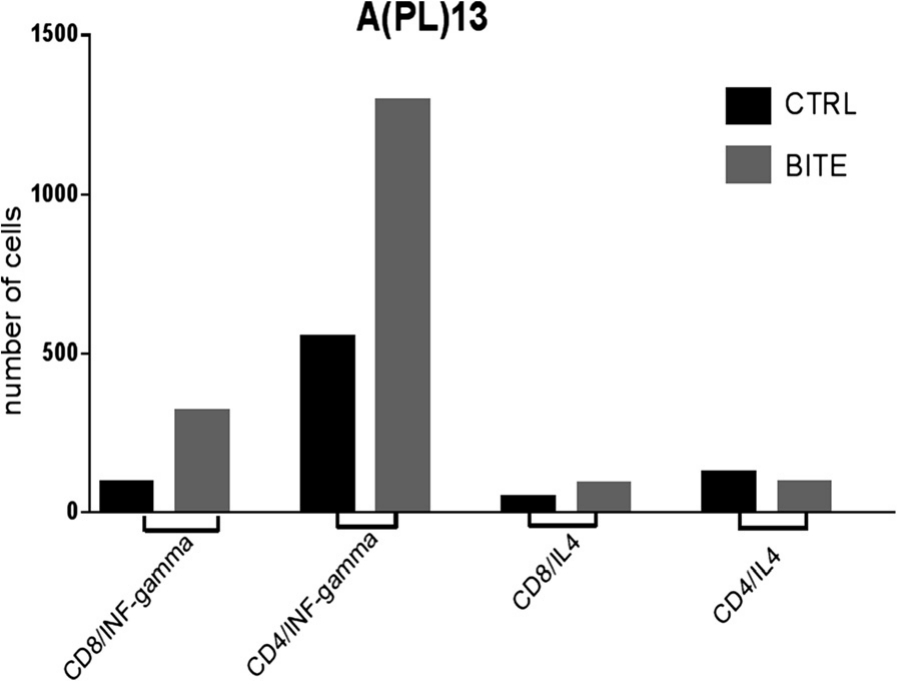
Representative IFN-gamma cytokine release after stimulation of T-lymphocytes exposed to solitomab vs control BiTE®. A significant increase in IFN-gamma stained cells was consistently detected in both CD8^+^ or CD4^+^ T cells (CD8/INF-gamma, CTRL VS BITE, *p* = 0.001478197; CD4/INF-gamma, CTRL VS BITE, *p* = 7.84945E-06; CD8/IL4, CTRL VS BITE, *p* not significant; CD4/IL4, CTRL VS BITE p not significant)

Next we investigated the level of the solitomab dependent cytokine induction in PBL when compared to the cytokine release of PBL stimulated with non-specific BiTE® control wells in the presence of EpCAM positive CS. The flow analysis was performed after 7 days of co-incubation of solitomab with tumor cells and PBLs before lymphocyte stimulation in the presence of brefeldin A as described in the Methods section. The analysis by FACS revealed a significant increase in IFN-gamma production in EpCAM + pleuric fluid exposed to solitomab when compared to control BiTE® wells in both CD8+ T and CD4+ T cells, (Fig. 4). In contrast, we were unable to demonstrate any increase in IL-4 cytokine release in solitomab exposed pleural fluid T-cells when compared to wells stimulated with control BiTE®.

## Discussion

Carcinosarcomas of gynecologic origin are rare and comprise 2–5 % of uterine and 1–2 % of ovarian cancers [1–3]. Although surgical debulking followed by chemotherapy is the mainstay of treatment for both uterine and ovarian CS, the overall 5-year survival is 30 ± 9 % for all stages, and the recurrence rate after treatment is extremely high (50–80 %) (1–3). The atypical pathogenesis and poor prognosis of uterine and ovarian CS, despite aggressive conventional treatment modalities, have inspired us to better understand the molecular basis of CS in hope of developing novel and effective treatment modalities.

EpCAM expression has been reported in a number of different carcinomas [4–7, 11–13]. EpCAM expression in nonmalignant tissue is low and mostly limited to the basolateral surface of epithelia [4–7, 11–13]. The physiologic location and low level expression of EpCAM in normal tissues allow for minimal interaction between these proteins and intravenously administered anti-EpCAM mAb. EpCAM mAb accessibility data gathered from studies using mice harboring human xenografts of EpCAM-expressing syngeneic tumors and normal epithelial tissues support this idea [13]. The differential expression and accessibility of EpCAM on metastatic/chemotherapy-resistant CS cells compared to normal tissues make EpCAM an attractive protein that might be exploited for targeted therapy. As a result patients with EpCAM expressing carcinosarcomas refractory to standard treatments maybe benefit from BiTE®-based immunotherapy.

BiTE® molecules act through linking tumor cells expressing EpCAM to CD3 T cell receptors with single-chain bispecific antibody constructs. Directly linking CD3 T cell-receptors to tumor cells allow for direct T cell stimulation and response. This direct response is not restricted by presence of MHC I, T cell receptor specificity, antigen presentation, or T cell co-stimulation. Data presented here support the potential of solitomab, an EpCAM/CD3 bispecific antibody construct (BiTE®), as a new therapeutic strategy against chemotherapy-resistant EpCAM expressing CS.

Cell lines established from patients harboring advanced and/or chemotherapy resistant carcinosarcoma were assessed for EpCAM expression as were free tumor cells and spheroids collected via thoracocentesis from a patient. EpCAM was overexpressed in the majority of the primary CS cell lines. These data are similar to data we previously reported for EpCAM in ovarian cancer patients [9, 14] as well as TROP-2, another cell-surface glycoprotein and potential target in CS [15]. Furthermore CS cell lines expressing EpCAM were susceptible to ADCC when exposed to solitomab. Studies previously demonstrated the cytotoxic activity of solitomab against a human colon cancer cell line as well as metastatic ovarian cancer cells in the presence of PBL [10, 16]. The data presented here suggest that the cytotoxic potential of solitomab may be extended to biologically aggressive and/or highly chemotherapy-resistant to uterine and ovarian CS cell lines. Solitomab-mediated T cell killing in the presence of effector cells was dependent on EpCAM surface expression. Tumor cells were highly susceptible to T cell mediated killing if EpCAM was present while negligible cytotoxicity was seen in cell lines with low level of EpCAM expression.

NK cells are known to play a critical role in the elimination and surveillance of cancer. Interestingly, EpCAM-positive CS cell lines were highly resistant to natural killer cytotoxic activity. While in the present study we did not investigate the mechanisms of resistance to NK cells, efficacy of cancer immunotherapy with antibodies activating cellular and complement-mediated cytotoxicity is known to be limited by the overexpression of multiple membrane-bound complement-regulatory protein (mCRPs). Consistent with this view, recent data from our group in uterine serous carcinomas, a subset of biologically aggressive tumors histologically similar to CS, demonstrate overexpression of mCRPs CD46, CD55 and CD59 in the majority of the tumors [17].

Importantly, primary CS cell lines in our study were highly sensitive to solitomab-mediated T cell cytotoxicity in 4-h ^51^Cr release assays. Data also show that ex vivo CS single cell suspensions and tumor spheroids isolated from the pleural fluid of a CS patient harboring chemotherapy resistant disease may also be highly sensitive to solitomab-mediated cytotoxicity. These results were obtained by culturing freshly collected tumor and tumor associated lymphocytes ex vivo. After adding solitomab to the culture, without immune-stimulatory cytokines, tumor killing was observed. These data suggest that solitomab may be an efficacious treatment for recurrent disease *in vivo*.

Our data suggests that solitomab is a potent stimulator of T cell cytotoxicity even at low effector to target ratios while simultaneously inducing significant CD8+ and CD4+ T lymphocyte proliferation. TAL isolated from the pleural fluid of a heavily pretreated CS patient, and subsequently exposed to solitomab, increase their production of type I cytokine production. The integrity and size of CS tumor spheroids, found in ex vivo cultures containing tumor cells expressing EpCAM and autologous tumor associated lymphocytes, were diminished after exposure to solitomab. Previous studies have suggested that TIL and TAL associated with tumors or ascitic fluids are frequently anergic and/or tolerogenic towards autologous tumor cells [18–21]. The addition of solitomab to cultures of EpCAM expressing CS and autologous TAL appears to activate these tumor-anergic T cells in pleural fluid causing a cytotoxic T-cell response strong enough to eliminate resistant CS cells.

Solitomab, and other antibody/BiTE®-based therapies, may be effective through either IV or intraperitoneal/intrapleural administration because of the frequency of expression of EpCAM on the surface of CS combined with its minimal expression on mesothelial type cells in the abdominal or pleural cavity. EpCAM/CD3-bispecific antibody construct (MT110) is currently being explored in phase 1 studies of patients harboring advanced stage tumors [22]. A trifunctional anti-EpCAM antibody (catumaxomab/Removab®), administered intraperitoneally to patients harboring ovarian carcinomas refractory to salvage chemotherapy, decreases tumor burden, ascites accumulation, and necessity of paracentesis [23]. These data resulted in the approval of catumaxomab in Europe for the treatment of chemotherapy refractory ovarian cancer. A drug with similar format as solitomab, blinatumomab, has been approved in the US for the treatment of Philadelphia chromosome-negative relapsed or refractory acute lymphoblastic leukemia. Like solitomab, blinatumomab activates T cell function through binding of CD3 and is a bispecific antibody construct that is a CD19-directed CD3 T-cell engager (BiTE®) [24–26].

Ovarian and uterine carcinosarcoma are aggressive tumors that are difficult to treat initially and particularly in the recurrent state. The data presented here demonstrate that EpCAM is expressed in the majority of carcinosarcomas evaluated (80 %) and that solitomab effectively induced ADCC and significant tumor death in carcinosarcoma cell lines expressing EpCAM that were incubated with PBL. Solitomab, compared to control BiTE®, caused an increase in the production of type 1 cytokines and cytotoxic activity of tumor associated lymphocytes isolated from pleural fluid in CS harboring patients and may increase recruitment as well as activation of CD4 and CD8+ T cells. Clinical trials should be designed to assess the efficacy of solitomab as a novel therapeutic approach in patients with chemotherapy refractory carcinosarcoma.

## Abbreviations

CS: Carcinosarcomas
EpCAM: Epithelial-cell-adhesion-molecule
ADCC: Antibody-dependent-cellular-cytotoxicity
TAL: Tumor-associated-T cells
PBL: Peripheral blood lymphocytes
PCR: Polymerase chain reaction
qRT-PCR: Quantitative real-time polymerase chain reaction
SEM: Standard error of the mean
FITC: Fluorescein isothiocyanate
MFI: Mean fluorescent intensity
OS: Overall survival

## References

[1] Schipf A, Mayr D, Kirchner T, Diebold J (2008). Molecular genetic aberrations of ovarian and uterine carcinosarcomas--a cgh and fish study. Virchows Arch..

[2] Cantrell LA, Van Le L (2009). Carcinosarcoma of the ovary a review. Obstet Gynecol Surv..

[3] Garg G, Shah JP, Kumar S, Bryant CS, Munkarah A, Morris RT (2010). Ovarian and uterine carcinosarcomas: A comparative analysis of prognostic variables and survival outcomes. Int J Gynecol Cancer..

[4] Choijamts B, Jimi S, Kondo T, Naganuma Y, Matsumoto T, Kuroki M (2011). Cd133+ cancer stem cell-like cells derived from uterine carcinosarcoma (malignant mixed mullerian tumor). Stem cells.

[5] Pietzner K, Woopen H, Richter R, Joens T, Braicu EI, Dimitrova D (2013). Expression of epithelial cell adhesion molecule in paired tumor samples of patients with primary and recurrent serous ovarian cancer. Int J Gynecol Cancer..

[6] Munz M, Baeuerle PA, Gires O (2009). The emerging role of epcam in cancer and stem cell signaling. Cancer Res..

[7] Armstrong A, Eck SL (2003). Epcam: A new therapeutic target for an old cancer antigen. Cancer Biol Ther..

[8] Brischwein K, Schlereth B, Guller B, Steiger C, Wolf A, Lutterbuese R (2006). Mt110: A novel bispecific single-chain antibody construct with high efficacy in eradicating established tumors. Mol Immunol..

[9] Bellone S, Siegel ER, Cocco E, Cargnelutti M, Silasi DA, Azodi M (2009). Overexpression of epithelial cell adhesion molecule in primary, metastatic, and recurrent/chemotherapy-resistant epithelial ovarian cancer: Implications for epithelial cell adhesion molecule-specific immunotherapy. Int J Gynecol Cancer..

[10] English DP, Bellone S, Schwab CL, Roque DM, Lopez S, Bortolomai I (2015). Solitomab, an epithelial cell adhesion molecule/cd3 bispecific antibody (BiTE), is highly active against primary chemotherapy-resistant ovarian cancer cell lines in vitro and fresh tumor cells ex vivo. Cancer..

[11] Balzar M, Winter MJ, de Boer CJ, Litvinov SV (1999). The biology of the 17-1A antigen (Ep-CAM). J Mol Med (Berl).

[12] Spurr NK, Durbin H, Sheer D, Parkar M, Bobrow L, Bodmer WF (1986). Characterization and chromosomal assignment of a human cell surface antigen defined by the monoclonal antibody AUAI. Int J Cancer..

[13] McLaughlin PM, Harmsen MC, Dokter WH, Kroesen BJ, van der Molen H, Brinker MG (2001). The epithelial glycoprotein 2 (EGP-2) promoter-driven epithelial-specific expression of EGP-2 in transgenic mice: a new model to study carcinoma-directed immunotherapy. Cancer Res..

[14] Richter CE, Cocco E, Bellone S, Silasi DA, Rüttinger D, Azodi M (2010). High-grade, chemotherapy-resistant ovarian carcinomas overexpress epithelial cell adhesion molecule (EpCAM) and are highly sensitive to immunotherapy with MT201, a fully human monoclonal anti-EpCAM antibody. Am J Obstet Gynecol.

[15] Raji R, Guzzo F, Carrara L, Varughese J, Cocco E, Bellone S (2011). Uterine and ovarian carcinosarcomas overexpressing Trop-2 are sensitive to hRS7, a humanized anti-Trop-2 antibody. J Exp Clin Cancer Res.

[16] Schlereth B, Fichtner I, Lorenczewski G, Kleindienst P, Brischwein K, da Silva A (2005). Eradication of tumors from a human colon cancer cell line and from ovarian cancer metastases in immunodeficient mice by a single-chain Ep-CAM-/CD3-bispecific antibody construct. Cancer Res..

[17] Bellone S, Roque D, Cocco E, Gasparrini S, Bortolomai I, Buza N (2012). Downregulation of membrane complement inhibitors CD55 and CD59 by siRNA sensitises uterine serous carcinoma overexpressing Her2/neu to complement and antibody-dependent cell cytotoxicity in vitro: implications for trastuzumab-based immunotherapy. Br J Cancer.

[18] Radoja S, Saio M, Schaer D, Koneru M, Vukmanovic S, Frey AB (2001). CD8(+) tumor-infiltrating T cells are deficient in perforin-mediated cytolytic activity due to defective microtubule-organizing center mobilization and lytic granule exocytosis. J Immunol..

[19] Bocchia M, Bronte V, Colombo MP, De Vincentiis A, Di Nicola M, Forni G (2000). Antitumor vaccination: where we stand. Haematologica..

[20] Costello RT, Gastaut JA, Olive D (1999). Tumor escape from immune surveillance. Arch Immunol Ther Exp (Warsz).

[21] Sinkovics JG, Horvath JC (2000). Vaccination against human cancers. Int J Oncol..

[22] Walter M, Fiedler MW, Maxim Kebenko, Marie-Elisabeth Goebeler, et al. A phase I study of EpCAM/CD3-bispecific antibody (MT110) in patients with advanced solid tumors. Oral Abstract Session, Developmental Therapeutics - Clinical Pharmacology and Immunotherapy Oral Abstract Session 2012 ASCO Annual Meeting.

[23] Burges A, Wimberger P, Kumper C, Gorbounova V, Sommer H, Schmalfeldt B (2007). Effective relief of malignant ascites in patients with advanced ovarian cancer by a trifunctional anti-EpCAM x anti-CD3 antibody: a phase I/II study. Clin Cancer Res..

[24] Topp MS, Gokbuget N, Zugmaier G, Klappers P, Stelljes M, Neumann S (2014). Phase II trial of the anti-CD19 bispecific T cell-engager blinatumomab shows hematologic and molecular remissions in patients with relapsed or refractory B-precursor acute lymphoblastic leukemia. J Clin Oncol..

[25] Sanford M (2015). Blinatumomab: first global approval. Drugs.

[26] Oak E, Bartlett NL. Blinatumomab for the treatment of B-cell lymphoma. Expert Opin Investig Drugs. 2015;24:715-24. 10.1517/13543784.2015.1021415 25739952

